# LDHA gene is associated with pigeon survivability during racing competitions

**DOI:** 10.1371/journal.pone.0195121

**Published:** 2018-05-18

**Authors:** Sherif Ramadan, Takeshi Miyake, Junichi Yamaura, Miho Inoue-Murayama

**Affiliations:** 1 Wildlife Research Center, Kyoto University, Kyoto, Japan; 2 Animal Wealth Development Department, Faculty of Veterinary Medicine, Benha University, Moshtohor, Egypt; 3 Graduate School of Agriculture, Kyoto University, Kyoto, Japan; 4 Japan Racing Pigeon Association, Kashiwa, Japan; 5 Wildlife Genome Collaborative Research Group, National Institute for Environmental Studies, Tsukuba, Japan; College of Agricultural Sciences, UNITED STATES

## Abstract

Pigeon racing is a popular sport worldwide. Pigeons are under continuous selection to improve speed, spatial orientation, and endurance during long flights. However, numerous genetic and non-genetic factors affect survivability and homing ability, making such traits difficult for breeders to control. Polymorphisms in the lactate dehydrogenase A gene (*LDHA*) likely affects pigeon racing and homing abilities, due to its role in physical and mental performance. Additionally, the adenylate cyclase activating polypeptide 1 gene (*ADCYAP1*) has been associated with physiological and behavioral shifts that occur during avian migration. In this study, we examined the association between *LDHA* and *ADCYAP1* genotypes with pigeon survivability during racing competitions. Survivability was evaluated through the estimated breeding value (EBV) of each individual’s total race distances during its athletic life. *ADCYAP1* was not polymorphic among our samples, while *LDHA* genotypes were significantly associated with deviated EBV values of longer total race distance; individuals carrying the *S+* genotype had higher EBV (i.e., greater survivability). Thus, the *LDHA* locus might be useful for marker-assisted selection, empowering breeders and trainers to maximize pigeon quality. Moreover, data obtained from breeding will also improve our understanding of the genetic mechanism underlying navigation and flight for wild migrating bird species.

## Introduction

Since the domestication of wild rock pigeons (*Columba livia*), breeders has been using artificial selection for generating pigeons with preferred appearances and improved homing ability [1, 2]. Such pigeons have historically been used for a variety of tasks, including the sport of pigeon racing, now popular globally [3, 4]. The breeding of suitable racing pigeons is lucrative, with a price of $328,000 for an individual in 2012 [5]. The distances of major races in Japan range from 100 km to 1000 km, while international races, such as Diamond Elite and Golden Island One-loft in China, are 300 miles and 310 miles, respectively [6]. Thus, racing pigeons are under intensive selection for improving of speed, endurance during long flights [7], along with general survivability and homing ability. Unfortunately, the latter two traits are controlled by various genetic and non-genetic factors, leading to considerable difficulties in breeding to enhance them. For instance, racing pigeons encounter numerous environmental hazards during racing competitions that greatly affect survivability and successful completion of the race, with predation by wild raptors being a primary hazard. Peregrine falcons, a common predator, mainly kill racing pigeons during flight when they are away from the loft area or during exercise flights [8–10]. Other external environmental conditions that negatively influence survivability include poor weather that disorient pigeons, collisions with solid objects (e.g., buildings, windows, vehicles, electricity wires), and entanglement in netting. Intrinsic factors that affect survivability may include straying, insufficient desire/motivation to return, exhaustion, lack of intelligence, and poor homing ability [11, 12].

Genetic markers have been used to predict racing performance in humans [13], horses [14], dogs [15], and pigeons [6, 16, 17]. These studies have indicated that lactate dehydrogenase isoform A, encoded by the lactate dehydrogenase A gene (*LDHA*), is critical to the aerobic/anaerobic metabolism of different tissues and thus to overall physiological performance. LDHA catalyzes the interconversion of pyruvate and lactate, with nicotinamide adenine dinucleotide as a coenzyme [18–20]. Lactate in particular is as an important cellular fuel, including for hippocampal nerve cells. Lactate’s function is now considered similar to a hormone, involved in processes as complex as memory formation and spatial working memory [21, 22]. Therefore, *LDHA* polymorphisms can potentially affect pigeon homing and racing abilities [6, 16, 23].

Another important gene likely to affect pigeon performance is the adenylate cyclase activating polypeptide 1 gene (*ADCYAP1*), which encodes pituitary adenylate cyclase-activating polypeptide (PACAP). *ADCYAP1* polymorphisms influence clock gene expression and thus plays a role in nocturnal migratory restlessness [24]. During migration season, nocturnal migrating birds maintain high physical and cognitive functions at times when they usually sleep; this manifests as prolonged flight, as well as enhanced navigation ability, survivability, and alertness against predators [25]. In blackcaps (*Sylvia atricapilla*), a significant association was observed between a microsatellite locus located in the 3′ UTR of *ADCYAP1* and migratory restlessness [24]. Previous behavioral experiments have demonstrated that pigeons exhibit nocturnal homing behavior [26].

Various measures such as race time, lifetime earnings, ranking and accumulated distance are used for evaluating racing ability [27]. An estimated breeding value (EBV) can be calculated for the previous traits to express their degree of heredity [28]. The EBV is more suitable than direct phenotypic measurements for association studies [28, 29]. Here, we evaluated individual pigeon survivability by calculating EBV based on accumulated total distances in each bird’s athletic lifetime. The aim of this study was to analyze the association between *LDHA* and *ADCYAP1* polymorphisms with survivability, as represented by the calculated EBV.

## Materials and methods

### Sample collection and DNA extraction

Feather samples (*n* = 127) of Japanese racing pigeons in Chiba, Japan were collected from five pigeon breeders belonging to the Japan Racing Pigeon Association. All analyzed pigeons were from the same population, maintained by a former Japanese army. We also note that the five breeders involved are acquainted with one another exchanging management and breeding experiences with each other and enter their pigeons in similar races. Therefore, no population stratification is apparent in the data. DNA was extracted from feather roots using the QIAGEN DNeasy Tissue Kit (QIAGEN, Valencia, CA, USA).

### Genotyping

The microsatellites of *LDHA* and *ADCYAP1* were amplified using fluorescently labeled (6-FAM) forward primers (Table 1). PCR reaction mixture was 10 μL, including 20 ng of genomic DNA, 2x GC buffer I, dNTPs at 400 μM each, forward/reverse primers at 0.3 μM, and 0.5 U of *LA-Taq* DNA polymerase (TaKaRa, Shiga, Japan). Thermocycling conditions were: denaturing 94°C for 1 min; 35 cycles of 94°C for 30 sec, 55°C for 45 sec, 74°C for 30 sec for *LDHA* while 94°C for 30 s, 55°C for 30 s, 74°C for 1 min for *ADCYAP1*; and final extension at 74°C for 10 min. PCR products were electrophoresed on an ABI 3130xl DNA Sequencer (Applied Biosystems, Foster City, CA, USA). Fragment sizes were estimated based on the fluorescently labeled forward primer in GENEMAPPER (Applied Biosystems, Foster City, CA, USA).

**Table 1:** Primer sequences and target microsatellites.

Gene (position)	Microsatellite	Primer sequence	Reference
*LDHA* (intron 6)	(TTTAT)_3-5_	F 5′-CCTGAAGGCTCTTCATCCAG-3′	16
		R 5′-TTGGGTGCACTCTTCTCAAA-3′	
*ADCYAP1*(3′-UTR)	(GA)_12_AAA(GA)_7_	F 5′-GATGTGAGTAACCAGCCACT- 3′	This study
		R 5′-ATAACACAGGAGCGGTGA- 3′	

### Statistical analysis

#### Categorizations of accumulated total race distances

To investigate the genetic performance of racing pigeons, we generated categorical binary phenotypes based on accumulated total race distances (TD) of 867 pedigreed Japanese racing pigeons during their athletic life. These binary phenotypes were analysed using categorical best linear unbiased predictions (BLUP) [30] to obtain EBVs of the pigeons. Five thresholds were artificially chosen (500, 1000, 2000, 3000, and 4000 km) to divide TD, resulting in five categorical binary traits: Race1 (Dist 500), Race2 (Dist 1000), Race3 (Dist 2000), Race4 (Dist 3000) and Race5 (Dist 4000), respectively (S1 Table). This categorization allowed us to investigate genetic variation underlying the race-distance phenotype. Higher TD indicates enhanced survivability and homing ability.

#### Heritability and estimated breeding value (EBV) estimation

To estimate the heritability and EBV for the five categorical race traits (Race1 to Race5), 867 Japanese racing pigeons were used. Subjects were born during 1989–2012 and registered with the Japan Racing Pigeon Association. Their pedigrees were traced back for five generations, resulting in 2,037 pedigrees total. Next, EBVs were estimated for the pedigree containing the 127 genotyped pigeons [30, 31] and used for the association study. This pigeon population is the same as the birds investigated in our previous study [16].

The five race traits were analysed using non-linear categorical (threshold) trait models with the GSTM (Gibbs Sampling Threshold Model) program [32], based on Sorensen *et al*. [31]. This program has been used for genetic analyses of several categorical traits in guide dogs and thoroughbred horses [27, 33, 34]. The following model was used to perform a non-linear categorical trait analysis:

$$L_{ij}=SEX_{i}+u_{j}+e_{ij}$$

Where *L*_*ij*_ is the normally distributed liability assumed with threshold *t*. The categorical phenotype values of *Y*_*ij*_ begin with 1 [31]. For example, the relationship between *L*_*ij*_, *Y*_*ij*_, and *t* is as follows for binary phenotypes: *Y*_*ij*_ = 1 (*L*_*ij*_ ≤ *t*) and *Y*_*ij*_ = 2 (*t* < *L*_*ij*_). Here, *t* represents the five race traits (500 km, 1000 km, 2000 km, 3000 km, and 4000 km). *SEX*_*i*_ is the effect of the *i*th sex. *u*_*j*_ is the breeding value of the *j*^th^ individual, following normal distribution [*N*(0, Aσ^2^_*u*_)]. Next, *e*_*ij*_ is the residual effect [*N*(0, Iσ^2^_*e*_)], A is the numerator relationship matrix in BLUP [30], σ^2^_*u*_ is the additive genetic variance, and σ^2^_*e*_ is the residual variance. Under this model with *L*_*ij*_, EBVs are also based on a normal distribution like *L*_*ij*_. Heritability (*h*^*2*^) is then defined as σ^2^_*u*_ / (σ^2^_*u*_ + σ^2^_*e*_).

The total number of Gibbs samplings in GSTM was 5,050,000 with a burn-in period of 50,000 and spacing of 2,500; these values are sufficiently large for parameter convergence. Finally, the deviations of EBVs were calculated with an average of 50.0 and a standard deviation of 10.0, and then used for the association study.

A generalized linear model (GLM) was used for the association analysis between *LDHA* genotypes with deviated EBV values (DEBV), in SAS version 9.1.3 (SAS Institute Inc., Cary, NC, USA). Because *S/S* and *S/L* genotypes were low in frequency, we combined all genotypes containing the minor allele (*S*) as the *S+* genotype (*S+* = *S/S*, *S/M*, *S/*L), while the remainder were categorized as *S*- genotype (*S-* = *M/M*, *M/L*, *L/L*).

#### Ethical statements

All aspects of the study were performed according to the guidelines established by the Ministry of Education, Culture, Sports, Science, and Technology in Japan (Notice No. 71). The protocol was approved by the Committee on the Ethics of Animal Experiments of the Wildlife Research Center of Kyoto University (Permit No. WRC-2017-002A).

## Results

The five race traits exhibited moderate to high heritability estimates (*h*^*2*^): 0.23, 0.25, 0.39, 0.28, and 0.20 for Race1, Race2, Race3, Race4, and Race5, respectively. Thus, the five complex traits of interest are heritable, with influence from both genetic and non-genetic factors.

We amplified and sequenced a 155 bp DNA fragment that includes the (GA)_12_AAA(GA)_7_ microsatellite in 3′ UTR of *ADCYAP1* (GenBank accession number: MF099650). Subjects were not polymorphic at this microsatellite locus. For *LDHA*, genotyping the microsatellite (TTTAT)_3-5_ in intron 6 yielded three alleles (*S*, 595 bp; *M*, 600 bp; *L*, 605 bp) and six genotypes (*S/S*, *S/M*, *S/L*, *M/M*, *M/L*, *L/L*). This polymorphic site was previously described in intron 5 [16]. The positional discrepancy was due to an update of the *LDHA* sequence that added a first non-coding exon (GenBank, Gene ID: 102094829).

**Fig 1:**
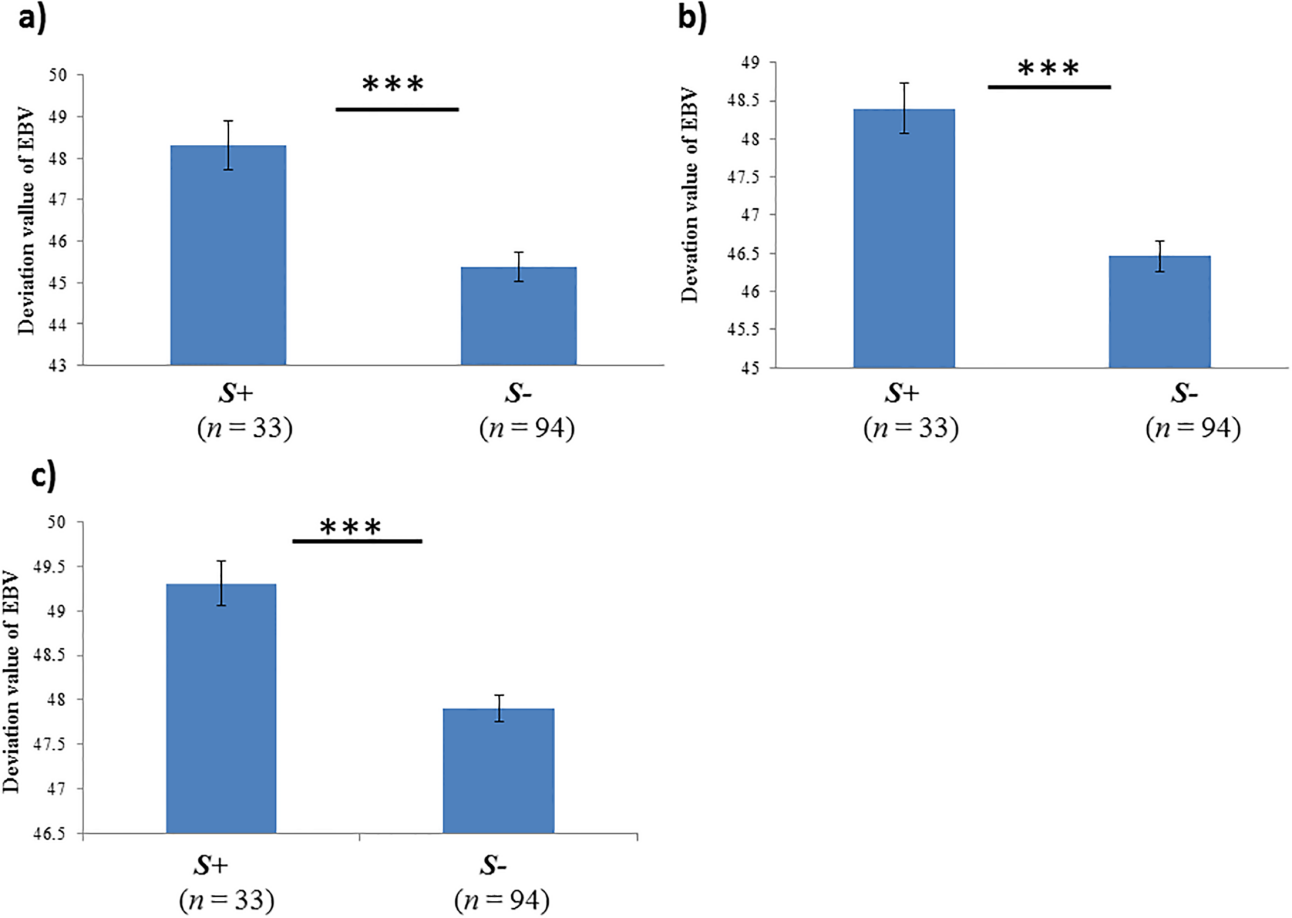
*LDHA* genotypes (existence of allele *S*) and deviations of estimated breeding value (DEBV) under different race traits. a) Race 3, b) Race 4, and c) Race 5.

The *M* allele (0.614) and *M/M* genotype (0.354) were highest in frequency, while the *S* allele (0.146) and *S/L* genotype (0.024) were the lowest (Table 2). The highest and lowest mean DEBVs were recorded for Race5 (48.266) and Race3 (46.134), respectively (Table 3; for EBVs and DEBVs per 127 genotypes, see S2 Table). *LDHA* genotypes were significantly associated with the DEBVs of three race traits (Race3–5). Specifically, *S*+ individuals exhibited increased survivability (higher DEBV) during racing competitions (Table 4; Fig 1).

**Table 2:** Observed numbers and frequencies of *LDHA* alleles and genotypes.

	Allele frequency			Genotype frequency							
Allele/genotype	*S*	*M*	*L*	*S/S*	*S/M*	*S/L*	*M/M*	*M/L*	*L/L*	*S+*	*S-*
Number	37	156	61	4	26	3	45	40	9	33	94
Frequency	0.146	0.614	0.240	0.031	0.205	0.024	0.354	0.315	0.071	0.260	0.740

The *LDHA* locus deviated from Hardy Weinberg equilibrium (*P* < 0.001)

**Table 3:** Mean, minimum, and maximum deviations of DEBV for five racing traits of 127 racing pigeons.

Traits	Mean ± SD	Minimum	Maximum
Race1 (Dist 500 km)	48.103±6.026	44.224	52.746
Race2 (Dist 1000 km)	46.946±7.923	42.501	53.705
Race3 (Dist 2000 km)	46.134±11.367	41.370	57.445
Race4 (Dist 3000 km)	46.965±7.493	43.662	54.258
Race5 (Dist 4000 km)	48.266±5.884	45.840	54.161

**Table 4:** Effect of *LDHA* genotypes (presence of allele *S*) on the five studied racing traits.

Racing traits	*P* value
Race1 (Dist 500 km)	0.987
Race2 (Dist 1000 km)	0.611
Race3 (Dist 2000 km)	0.001
Race4 (Dist 3000 km)	0.001
Race5 (Dist 4000 km)	0.001

## Discussion

In this study, we investigated the association between racing-pigeon survivability and polymorphisms in two genes, the novel *ADCYAP1* and the well-studied *LDHA*. Although we did not observe polymorphism in *ADCYAP1*, *LDHA* polymorphisms were significantly associated with DEBV: individuals carrying the *S+* genotype exhibited higher DEBV of three race-distance-related traits (Race3–5). The lack of significant relationships between *LDHA* genotypes and shorter race distances (Race1 and Race2) may be because pigeon survivability matters less at 500–1000 km. Our results corroborate multiple studies that have demonstrated a significant link between *LDHA* polymorphisms and pigeon racing/homing abilities [6, 16, 23]. These data are in line with existing knowledge of LDHA function. Through its role in the astrocyte-neuron lactate shuttle (ANLS), LDHA affects overall physical and mental performance. This pathway involves the metabolism of glucose to pyruvate in astrocytes (a type of hippocampal glial cell), followed by LDHA reduction of pyruvate to lactate, which is then released and taken up by nearby neurons as a fuel source [35]. The hippocampus has a well-known role in spatial navigation and long-term memory formation [36].

Higher *M* allele frequencies were found in Japanese racing (0.712) than in wild rock pigeons (0.334), a trend that is attributable to the long history of artificial selection for high speed and rapid return during racing competitions [16]. In the current study, *M* allele frequency was also higher than *S* allele frequency, but *S/S* individuals exhibited greater survivability (i.e., higher EBV). This outcome is likely due to our chosen trait of interest and a trade-off between selecting for speed versus selecting for survivability. Specifically, accumulated total race distance in a pigeon’s athletic life involves completing many races safely, a factor primarily reliant on survivability. High survivability indicates excellent navigation ability, heightened endurance, and increased resistance against environmental hazards (e.g.., wild predators and bad weather), but not necessarily high speed. Wild rock pigeons have greater survivability (suggesting higher *S* frequency) than racing pigeons, given intense natural selective pressure on the former for traits such as foraging, seeking shelter, and predator defense. However, intense and extended artificial selection for high speed in racing pigeons appears to have increased *M* allele frequency at the expense of *S* allele.

The moderate to high *h*^*2*^ values for our five pigeon race traits were in agreement with previous reports on horse racing performance, which exhibited *h*^*2*^ values of 0.05–0.29 [27], 0.12–0.25 [37], and 0.11–0.25 [27, 37, 38]. However, our *h*^*2*^ estimates were higher than the estimate (0.06) from an earlier study on racing pigeons [4]. This considerable variation is likely due to the numerous factors that influence the heritability estimate, including species, populations, trait type, and trait measurement methods [27]. In the case of this study, our pigeon population descended from a group raised by the Japanese army and therefore no population stratification was present in the data. The test population exhibited a minor frequency of segregating *S* alleles, with occasional homozygotes that exhibit high survivability.

We can provide some hypotheses regarding how the microsatellite in *LDHA* intron 6 affects EBVs of accumulated total race distances. This polymorphic locus is near the GT splice donor site and thus may affects pre-mRNA splicing to influence *LDHA* gene expression. Alternatively, the microsatellite might be in linkage disequilibrium with some unknown functional locus located in close proximity to *LDHA*. Other important genes likely to affect pigeon performance are serotonin transporter (5-HTT) and mitochondrial ATP6 genes. 5-HTT affects personality and ATP6 affects energy production and physical fitness and both traits are important for survivability. We had surveyed *5-HTT* exon1 and *ATP6* among Japanese racing pigeons but we couldn’t find polymorphism within these two genes (data not shown). Future functional genomics and linkage analyses must include more candidate genes (e.g., dopamine receptor D4 [17] or *LDHA* coding exons [23]) and more racing pigeon populations to confirm the relationship between genetic variation and complex traits such as survivability. Nonetheless, the microsatellite locus reported here should prove useful for marker-assisted selection that can maximize the genetic quality of racing pigeons and will further our knowledge regarding the genetic underpinnings of survivability and navigation ability in wild migrating bird species.

## Supporting information

S1 Table Categorization of 867 racing pigeons for the five distance thresholds. (DOCX)

S2 Table *LDHA* genotypes for estimated breeding value (EBV) and deviations of EBV (DEBV) from 127 Japanese racing pigeons. (DOCX)
