# Supplementary material for: LDHA gene is associated with pigeon survivability during racing competitions
Source: PLoS One. 2018 May 18;13(5):e0195121. doi: 10.1371/journal.pone.0195121 (PMC5959059; doi:10.1371/journal.pone.0195121)
Supplement: S1 Table — (DOCX) [file pone.0195121.s001.docx]

**S1 Table**

| Distance threshold | Individuals lower than threshold | Individuals higher than Threshold |
| --- | --- | --- |
| 500 km  (Race 1) | 325 | 542 |
| 1000 km (Race 2) | 492 | 375 |
| 2000 km (Race 3) | 705 | 162 |
| 3000 km (Race 4) | 790 | 77 |
| 4000 km (Race 5) | 823 | 44 |
