# Supplementary material for: LDHA gene is associated with pigeon survivability during racing competitions
Source: PLoS One. 2018 May 18;13(5):e0195121. doi: 10.1371/journal.pone.0195121 (PMC5959059; doi:10.1371/journal.pone.0195121)
Supplement: S2 Table — (DOCX) [file pone.0195121.s002.docx]

**S2 Table**

| ID | LDHA Genotype | Race1 (EBV) | Race1 (DEBV) | Race2 (EBV) | Race2 (DEBV) | Race3 (EBV) | Race3 (DEBV) | Race4 (EBV) | Race4 (DEBV) | Race5 (EBV) | Race 5 (DEBV) |
| --- | --- | --- | --- | --- | --- | --- | --- | --- | --- | --- | --- |
| 1 | S/S | 0.09 | 50.86 | 0.37 | 53.70 | 0.74 | 57.45 | 0.30 | 52.99 | 0.00 | 50.01 |
| 2 | S/S | -0.14 | 48.57 | 0.06 | 50.56 | 0.64 | 56.44 | 0.38 | 53.82 | -0.11 | 48.94 |
| 3 | S/S | -0.34 | 46.65 | -0.18 | 48.19 | 0.24 | 52.37 | 0.06 | 50.55 | -0.10 | 49.04 |
| 4 | S/S | -0.34 | 46.58 | -0.21 | 47.88 | -0.01 | 49.87 | -0.02 | 49.84 | 0.10 | 50.95 |
| 5 | M/M | 0.27 | 52.75 | 0.28 | 52.77 | -0.31 | 46.86 | -0.16 | 48.38 | -0.08 | 49.19 |
| 6 | M/M | -0.50 | 45.00 | -0.58 | 44.24 | -0.32 | 46.84 | -0.24 | 47.60 | -0.18 | 48.19 |
| 7 | M/M | -0.35 | 46.48 | -0.20 | 48.04 | -0.31 | 46.89 | -0.41 | 45.87 | -0.19 | 48.08 |
| 8 | M/M | -0.40 | 45.98 | -0.19 | 48.06 | -0.12 | 48.80 | -0.43 | 45.65 | -0.21 | 47.95 |
| 9 | M/M | -0.05 | 49.52 | -0.32 | 46.85 | -0.45 | 45.52 | -0.32 | 46.80 | -0.10 | 49.03 |
| 10 | M/M | -0.03 | 49.67 | -0.23 | 47.74 | -0.35 | 46.54 | -0.16 | 48.44 | -0.04 | 49.64 |
| 11 | M/M | -0.47 | 45.32 | -0.49 | 45.07 | -0.11 | 48.91 | -0.24 | 47.62 | -0.17 | 48.30 |
| 12 | M/M | -0.26 | 47.40 | -0.30 | 46.96 | -0.46 | 45.45 | -0.58 | 44.22 | -0.31 | 46.91 |
| 13 | M/M | -0.48 | 45.22 | -0.28 | 47.21 | -0.25 | 47.53 | -0.42 | 45.77 | -0.21 | 47.90 |
| 14 | M/M | -0.43 | 45.73 | -0.64 | 43.56 | -0.57 | 44.26 | -0.33 | 46.74 | -0.21 | 47.92 |
| 15 | M/M | -0.57 | 44.27 | -0.32 | 46.81 | -0.52 | 44.78 | -0.32 | 46.76 | 0.02 | 50.25 |
| 16 | M/M | 0.11 | 51.06 | -0.07 | 49.33 | -0.52 | 44.80 | -0.31 | 46.86 | -0.13 | 48.74 |
| 17 | M/M | -0.09 | 49.14 | -0.11 | 48.86 | -0.68 | 43.24 | -0.54 | 44.58 | -0.36 | 46.37 |
| 18 | M/M | -0.04 | 49.60 | -0.04 | 49.59 | -0.70 | 43.03 | -0.55 | 44.55 | -0.37 | 46.32 |
| 19 | M/M | -0.16 | 48.39 | -0.17 | 48.30 | -0.36 | 46.36 | -0.28 | 47.15 | -0.12 | 48.79 |
| 20 | M/M | -0.45 | 45.51 | -0.31 | 46.90 | -0.04 | 49.63 | -0.40 | 46.00 | -0.11 | 48.87 |
| 21 | M/M | -0.41 | 45.95 | -0.40 | 46.00 | -0.27 | 47.32 | -0.40 | 46.00 | -0.10 | 48.97 |
| 22 | M/M | 0.15 | 51.45 | -0.02 | 49.82 | -0.48 | 45.23 | -0.20 | 47.99 | -0.05 | 49.46 |
| 23 | M/M | -0.04 | 49.59 | 0.00 | 50.03 | -0.24 | 47.58 | -0.05 | 49.45 | -0.04 | 49.61 |
| 24 | M/M | -0.06 | 49.40 | -0.25 | 47.55 | -0.76 | 42.42 | -0.40 | 45.98 | -0.29 | 47.10 |
| 25 | M/M | -0.28 | 47.15 | -0.31 | 46.90 | -0.42 | 45.83 | -0.32 | 46.81 | -0.10 | 49.03 |

**S2 Table continued**

| ID | LDHA Genotype | Race1 (EBV) | Race1 (DEBV) | Race2 (EBV) | Race2 (DEBV) | Race3 (EBV) | Race3 (DEBV) | Race4 (EBV) | Race4 (DEBV) | Race5 (EBV) | Race 5 (DEBV) |
| --- | --- | --- | --- | --- | --- | --- | --- | --- | --- | --- | --- |
| 26 | M/M | -0.05 | 49.46 | 0.09 | 50.93 | -0.02 | 49.80 | -0.01 | 49.88 | 0.10 | 51.00 |
| 27 | M/M | -0.08 | 49.24 | -0.10 | 48.99 | -0.66 | 43.39 | -0.56 | 44.43 | -0.37 | 46.29 |
| 28 | M/M | -0.15 | 48.51 | -0.12 | 48.81 | -0.01 | 49.90 | -0.38 | 46.21 | -0.38 | 46.21 |
| 29 | M/M | 0.03 | 50.27 | -0.30 | 46.95 | 0.08 | 50.78 | -0.17 | 48.25 | -0.11 | 48.92 |
| 30 | M/M | -0.25 | 47.54 | -0.31 | 46.93 | -0.69 | 43.11 | -0.56 | 44.36 | -0.37 | 46.25 |
| 31 | M/M | -0.58 | 44.22 | -0.37 | 46.27 | -0.13 | 48.75 | -0.42 | 45.77 | -0.16 | 48.38 |
| 32 | M/M | -0.56 | 44.44 | -0.45 | 45.52 | -0.16 | 48.44 | -0.25 | 47.47 | -0.18 | 48.19 |
| 33 | M/M | -0.04 | 49.63 | 0.06 | 50.61 | -0.38 | 46.18 | -0.20 | 47.99 | -0.09 | 49.13 |
| 34 | M/M | -0.20 | 48.00 | -0.17 | 48.26 | -0.73 | 42.72 | -0.56 | 44.44 | -0.36 | 46.42 |
| 35 | M/M | 0.01 | 50.08 | 0.04 | 50.36 | 0.13 | 51.30 | 0.04 | 50.39 | -0.37 | 46.30 |
| 36 | M/M | -0.54 | 44.59 | -0.34 | 46.61 | -0.17 | 48.27 | -0.43 | 45.70 | -0.17 | 48.29 |
| 37 | M/M | -0.15 | 48.52 | -0.67 | 43.27 | -0.74 | 42.65 | -0.51 | 44.94 | -0.30 | 47.00 |
| 38 | M/M | -0.51 | 44.90 | -0.26 | 47.42 | -0.23 | 47.69 | -0.27 | 47.29 | -0.01 | 49.85 |
| 39 | M/M | -0.36 | 46.39 | -0.62 | 43.81 | -0.44 | 45.63 | -0.28 | 47.16 | -0.09 | 49.05 |
| 40 | M/M | 0.06 | 50.60 | 0.11 | 51.07 | -0.40 | 46.03 | -0.16 | 48.39 | -0.18 | 48.21 |
| 41 | M/M | -0.22 | 47.77 | -0.30 | 47.01 | -0.64 | 43.63 | -0.53 | 44.74 | -0.37 | 46.34 |
| 42 | M/M | -0.48 | 45.18 | -0.65 | 43.52 | -0.83 | 41.69 | -0.50 | 45.00 | -0.34 | 46.59 |
| 43 | M/M | -0.32 | 46.78 | -0.25 | 47.48 | -0.22 | 47.83 | -0.09 | 49.11 | -0.03 | 49.70 |
| 44 | M/M | 0.04 | 50.45 | -0.69 | 43.12 | -0.77 | 42.29 | -0.53 | 44.71 | -0.29 | 47.14 |
| 45 | M/M | -0.22 | 47.77 | -0.13 | 48.70 | -0.30 | 47.04 | -0.29 | 47.11 | -0.29 | 47.12 |
| 46 | M/M | -0.50 | 45.03 | -0.35 | 46.49 | -0.56 | 44.37 | -0.47 | 45.33 | -0.17 | 48.30 |
| 47 | M/M | -0.43 | 45.75 | -0.54 | 44.57 | -0.48 | 45.23 | -0.27 | 47.27 | -0.11 | 48.93 |
| 48 | M/M | -0.54 | 44.65 | -0.44 | 45.60 | -0.46 | 45.45 | -0.50 | 45.00 | -0.27 | 47.26 |
| 49 | M/M | -0.35 | 46.53 | -0.28 | 47.17 | -0.06 | 49.41 | -0.02 | 49.77 | -0.12 | 48.79 |
| 50 | M/L | -0.45 | 45.54 | -0.41 | 45.94 | -0.05 | 49.54 | 0.00 | 50.03 | 0.12 | 51.23 |

**S2 Table continued**

| ID | LDHA Genotype | Race1 (EBV) | Race1 (DEBV) | Race2 (EBV) | Race2 (DEBV) | Race3 (EBV) | Race3 (DEBV) | Race4 (EBV) | Race4 (DEBV) | Race5 (EBV) | Race 5 (DEBV) |
| --- | --- | --- | --- | --- | --- | --- | --- | --- | --- | --- | --- |
| 51 | M/L | -0.16 | 48.42 | -0.09 | 49.15 | 0.33 | 53.31 | -0.29 | 47.06 | -0.14 | 48.60 |
| 52 | M/L | -0.33 | 46.66 | -0.29 | 47.11 | -0.14 | 48.61 | -0.27 | 47.32 | -0.12 | 48.76 |
| 53 | M/L | -0.36 | 46.41 | -0.44 | 45.65 | -0.05 | 49.46 | 0.00 | 50.01 | 0.13 | 51.32 |
| 54 | M/L | -0.21 | 47.92 | -0.65 | 43.49 | -0.85 | 41.49 | -0.53 | 44.73 | -0.35 | 46.46 |
| 55 | M/L | 0.08 | 50.80 | -0.12 | 48.80 | 0.11 | 51.10 | -0.45 | 45.48 | -0.25 | 47.48 |
| 56 | M/L | -0.12 | 48.82 | -0.33 | 46.75 | -0.41 | 45.93 | -0.45 | 45.49 | -0.26 | 47.35 |
| 57 | M/L | -0.15 | 48.50 | -0.66 | 43.40 | -0.73 | 42.69 | -0.51 | 44.87 | -0.29 | 47.11 |
| 58 | M/L | -0.43 | 45.72 | -0.63 | 43.66 | -0.80 | 41.98 | -0.51 | 44.93 | -0.35 | 46.54 |
| 59 | M/L | -0.23 | 47.65 | -0.50 | 45.00 | -0.85 | 41.47 | -0.48 | 45.18 | -0.33 | 46.68 |
| 60 | M/L | 0.06 | 50.56 | -0.69 | 43.15 | -0.76 | 42.40 | -0.52 | 44.85 | -0.29 | 47.07 |
| 61 | M/L | -0.46 | 45.41 | -0.61 | 43.89 | -0.85 | 41.50 | -0.51 | 44.93 | -0.38 | 46.25 |
| 62 | M/L | 0.10 | 50.96 | -0.44 | 45.63 | -0.70 | 42.99 | -0.39 | 46.15 | -0.32 | 46.80 |
| 63 | M/L | -0.40 | 45.97 | -0.52 | 44.82 | -0.51 | 44.92 | -0.26 | 47.36 | -0.11 | 48.90 |
| 64 | M/L | -0.15 | 48.46 | -0.41 | 45.87 | -0.67 | 43.31 | -0.48 | 45.19 | -0.30 | 46.96 |
| 65 | M/L | -0.16 | 48.41 | -0.24 | 47.60 | -0.55 | 44.48 | -0.32 | 46.80 | -0.12 | 48.76 |
| 66 | M/L | 0.13 | 51.30 | 0.09 | 50.94 | -0.42 | 45.84 | -0.28 | 47.17 | -0.11 | 48.88 |
| 67 | M/L | -0.25 | 47.49 | -0.23 | 47.70 | 0.09 | 50.92 | 0.05 | 50.52 | 0.15 | 51.48 |
| 68 | M/L | -0.28 | 47.24 | -0.36 | 46.43 | -0.02 | 49.83 | 0.01 | 50.07 | 0.14 | 51.39 |
| 69 | M/L | 0.09 | 50.89 | 0.07 | 50.71 | -0.49 | 45.13 | -0.25 | 47.47 | -0.10 | 49.03 |
| 70 | M/L | 0.06 | 50.64 | -0.07 | 49.30 | -0.71 | 42.88 | -0.36 | 46.35 | -0.32 | 46.81 |
| 71 | M/L | 0.16 | 51.60 | 0.04 | 50.37 | -0.50 | 44.97 | -0.19 | 48.12 | -0.32 | 46.76 |
| 72 | M/L | 0.16 | 51.65 | -0.14 | 48.58 | -0.85 | 41.53 | -0.42 | 45.77 | -0.35 | 46.50 |
| 73 | M/L | -0.01 | 49.91 | 0.15 | 51.53 | -0.40 | 45.95 | -0.17 | 48.30 | -0.09 | 49.11 |
| 74 | M/L | -0.06 | 49.43 | -0.18 | 48.19 | -0.79 | 42.09 | -0.49 | 45.14 | -0.42 | 45.84 |
| 75 | M/L | -0.02 | 49.85 | 0.07 | 50.67 | -0.07 | 49.30 | -0.04 | 49.62 | -0.02 | 49.76 |

**S2 Table continued**

| ID | LDHA Genotype | Race1 (EBV) | Race1 (DEBV) | Race2 (EBV) | Race2 (DEBV) | Race3 (EBV) | Race3 (DEBV) | Race4 (EBV) | Race4 (DEBV) | Race5 (EBV) | Race 5 (DEBV) |
| --- | --- | --- | --- | --- | --- | --- | --- | --- | --- | --- | --- |
| 76 | M/L | -0.12 | 48.82 | -0.55 | 44.52 | -0.79 | 42.06 | -0.63 | 43.66 | -0.41 | 45.94 |
| 77 | M/L | -0.29 | 47.10 | -0.63 | 43.73 | -0.81 | 41.88 | -0.43 | 45.75 | -0.29 | 47.06 |
| 78 | M/L | -0.06 | 49.41 | -0.46 | 45.43 | -0.75 | 42.50 | -0.39 | 46.10 | -0.29 | 47.09 |
| 79 | M/L | -0.29 | 47.10 | -0.33 | 46.67 | -0.67 | 43.33 | -0.55 | 44.54 | -0.37 | 46.33 |
| 80 | M/L | -0.26 | 47.43 | -0.47 | 45.29 | -0.75 | 42.49 | -0.39 | 46.09 | -0.28 | 47.18 |
| 81 | M/L | 0.10 | 50.95 | 0.19 | 51.94 | -0.45 | 45.55 | -0.30 | 47.02 | -0.19 | 48.07 |
| 82 | M/L | 0.08 | 50.77 | 0.03 | 50.25 | -0.27 | 47.34 | -0.32 | 46.80 | -0.22 | 47.77 |
| 83 | M/L | -0.35 | 46.49 | -0.52 | 44.76 | -0.78 | 42.21 | -0.58 | 44.15 | -0.39 | 46.14 |
| 84 | M/L | -0.07 | 49.33 | -0.58 | 44.17 | -0.51 | 44.86 | -0.54 | 44.60 | -0.29 | 47.06 |
| 85 | M/L | -0.29 | 47.13 | -0.25 | 47.46 | 0.26 | 52.64 | 0.03 | 50.28 | 0.14 | 51.37 |
| 86 | M/L | -0.18 | 48.22 | -0.18 | 48.18 | -0.51 | 44.95 | -0.57 | 44.33 | -0.38 | 46.23 |
| 87 | M/L | -0.35 | 46.45 | -0.65 | 43.55 | -0.86 | 41.45 | -0.51 | 44.85 | -0.34 | 46.64 |
| 88 | M/L | -0.42 | 45.82 | -0.63 | 43.75 | -0.83 | 41.67 | -0.50 | 45.01 | -0.34 | 46.56 |
| 89 | M/L | -0.11 | 48.95 | -0.75 | 42.50 | -0.78 | 42.24 | -0.56 | 44.40 | -0.30 | 46.98 |
| 90 | L/L | 0.07 | 50.74 | -0.11 | 48.87 | -0.53 | 44.66 | -0.37 | 46.33 | -0.31 | 46.92 |
| 91 | L/L | 0.14 | 51.40 | -0.14 | 48.58 | -0.56 | 44.43 | -0.35 | 46.51 | -0.22 | 47.83 |
| 92 | L/L | -0.25 | 47.46 | -0.73 | 42.69 | -0.86 | 41.37 | -0.55 | 44.53 | -0.35 | 46.48 |
| 93 | L/L | -0.39 | 46.06 | -0.62 | 43.81 | -0.83 | 41.67 | -0.51 | 44.91 | -0.34 | 46.57 |
| 94 | L/L | 0.06 | 50.56 | -0.50 | 44.96 | -0.73 | 42.68 | -0.44 | 45.55 | -0.28 | 47.16 |
| 95 | L/L | -0.30 | 47.02 | -0.43 | 45.71 | -0.83 | 41.66 | -0.50 | 45.05 | -0.37 | 46.27 |
| 96 | L/L | 0.02 | 50.22 | -0.53 | 44.69 | -0.77 | 42.28 | -0.44 | 45.61 | -0.29 | 47.11 |
| 97 | L/L | -0.20 | 47.96 | -0.53 | 44.70 | -0.52 | 44.82 | -0.27 | 47.32 | -0.11 | 48.87 |
| 98 | L/L | 0.06 | 50.57 | -0.42 | 45.80 | -0.66 | 43.44 | -0.49 | 45.14 | -0.31 | 46.94 |
| 99 | S/M | -0.05 | 49.47 | 0.04 | 50.40 | 0.53 | 55.32 | 0.43 | 54.26 | 0.42 | 54.16 |
| 100 | S/M | -0.05 | 49.55 | -0.60 | 44.04 | -0.50 | 44.99 | -0.26 | 47.36 | -0.10 | 49.03 |

**S2 Table continued**

| ID | LDHA Genotype | Race1 (EBV) | Race1 (DEBV) | Race2 (EBV) | Race2 (DEBV) | Race3 (EBV) | Race3 (DEBV) | Race4 (EBV) | Race4 (DEBV) | Race5 (EBV) | Race 5 (DEBV) |
| --- | --- | --- | --- | --- | --- | --- | --- | --- | --- | --- | --- |
| 101 | S/M | -0.28 | 47.23 | -0.03 | 49.72 | 0.13 | 51.27 | -0.20 | 48.05 | -0.03 | 49.66 |
| 102 | S/M | -0.57 | 44.27 | -0.63 | 43.71 | -0.32 | 46.78 | -0.25 | 47.45 | -0.18 | 48.25 |
| 103 | S/M | -0.52 | 44.82 | -0.52 | 44.81 | -0.02 | 49.77 | -0.09 | 49.11 | -0.18 | 48.17 |
| 104 | S/M | -0.23 | 47.68 | 0.00 | 50.00 | 0.17 | 51.70 | -0.04 | 49.56 | 0.08 | 50.78 |
| 105 | S/M | -0.16 | 48.38 | -0.16 | 48.37 | 0.02 | 50.17 | 0.05 | 50.53 | 0.15 | 51.53 |
| 106 | S/M | -0.34 | 46.63 | -0.22 | 47.78 | -0.29 | 47.07 | -0.18 | 48.16 | 0.01 | 50.05 |
| 107 | S/M | -0.28 | 47.25 | -0.10 | 49.03 | -0.30 | 46.97 | -0.21 | 47.94 | 0.00 | 50.03 |
| 108 | S/M | 0.04 | 50.43 | -0.14 | 48.64 | -0.64 | 43.55 | -0.36 | 46.36 | -0.31 | 46.87 |
| 109 | S/M | 0.11 | 51.08 | -0.17 | 48.35 | -0.55 | 44.52 | -0.32 | 46.80 | -0.13 | 48.66 |
| 110 | S/M | 0.02 | 50.17 | -0.26 | 47.44 | 0.03 | 50.34 | -0.12 | 48.85 | 0.15 | 51.51 |
| 111 | S/M | 0.07 | 50.66 | 0.22 | 52.17 | -0.30 | 46.98 | -0.08 | 49.25 | 0.08 | 50.75 |
| 112 | S/M | -0.56 | 44.43 | -0.51 | 44.88 | -0.36 | 46.41 | -0.25 | 47.54 | -0.10 | 49.02 |
| 113 | S/M | -0.14 | 48.63 | 0.06 | 50.63 | 0.65 | 56.53 | 0.08 | 50.77 | -0.09 | 49.05 |
| 114 | S/M | -0.44 | 45.58 | -0.36 | 46.35 | -0.20 | 47.97 | -0.14 | 48.56 | 0.00 | 50.01 |
| 115 | S/M | -0.03 | 49.68 | 0.01 | 50.14 | 0.65 | 56.48 | -0.16 | 48.35 | -0.12 | 48.78 |
| 116 | S/M | -0.08 | 49.16 | -0.44 | 45.60 | -0.31 | 46.93 | -0.17 | 48.30 | -0.08 | 49.18 |
| 117 | S/M | 0.06 | 50.55 | -0.70 | 43.05 | -0.65 | 43.50 | -0.38 | 46.19 | -0.14 | 48.56 |
| 118 | S/M | -0.51 | 44.93 | -0.25 | 47.48 | -0.21 | 47.89 | -0.27 | 47.30 | -0.01 | 49.85 |
| 119 | S/M | -0.17 | 48.34 | -0.67 | 43.33 | -0.74 | 42.61 | -0.51 | 44.94 | -0.28 | 47.25 |
| 120 | S/M | -0.38 | 46.17 | -0.31 | 46.88 | -0.50 | 44.96 | -0.31 | 46.86 | -0.19 | 48.09 |
| 121 | S/M | -0.39 | 46.14 | -0.30 | 46.97 | -0.51 | 44.94 | -0.33 | 46.66 | -0.19 | 48.10 |
| 122 | S/M | -0.08 | 49.15 | -0.38 | 46.18 | -0.21 | 47.89 | -0.08 | 49.17 | 0.04 | 50.40 |
| 123 | S/M | -0.32 | 46.82 | -0.39 | 46.14 | -0.24 | 47.64 | -0.06 | 49.39 | 0.03 | 50.34 |
| 124 | S/M | -0.52 | 44.80 | -0.58 | 44.18 | -0.29 | 47.11 | -0.23 | 47.73 | -0.16 | 48.37 |
| 125 | S/L | 0.05 | 50.50 | -0.69 | 43.06 | -0.76 | 42.43 | -0.50 | 44.96 | -0.29 | 47.14 |
| 126 | S/L | 0.04 | 50.37 | -0.70 | 43.04 | -0.77 | 42.26 | -0.53 | 44.69 | -0.27 | 47.28 |
| 127 | S/L | 0.05 | 50.47 | -0.70 | 42.98 | -0.73 | 42.70 | -0.51 | 44.89 | -0.27 | 47.29 |
